# Contemporary Network Proteomics and Its Requirements

**DOI:** 10.3390/biology3010022

**Published:** 2013-12-20

**Authors:** Wilson Wen Bin Goh, Limsoon Wong, Judy Chia Ghee Sng

**Affiliations:** 1 School of Computing, National University of Singapore, 13 Computing Drive, Singapore 117417, Singapore; E-Mail: wongls@comp.nus.edu.sg; 2 Department of Physiology, National University of Singapore, 2 Medical Drive, Singapore 117597, Singapore; E-Mail: judy_sng@sics.a-star.edu.sg; 3 Neuroepigenetics Laboratory, Growth, Development and Metabolism Programme, Singapore Institute for Clinical Sciences, Agency for Science and Technology (A*STAR), Singapore 117609, Singapore

**Keywords:** proteomics, networks, systems biology, bioinformatics

## Abstract

The integration of networks with genomics (network genomics) is a familiar field. Conventional network analysis takes advantage of the larger coverage and relative stability of gene expression measurements. Network proteomics on the other hand has to develop further on two critical factors: (1) expanded data coverage and consistency, and (2) suitable reference network libraries, and data mining from them. Concerning (1) we discuss several contemporary themes that can improve data quality, which in turn will boost the outcome of downstream network analysis. For (2), we focus on network analysis developments, specifically, the need for context-specific networks and essential considerations for localized network analysis.

## 1. Introduction

Proteomics is the investigation of all the proteins in a given system (identification and quantitation). Currently, the most prevalent platform for proteomics is the mass spectrometer (MS) and, for focus, will be the only type of proteomics discussed here. MS-based proteomics strategies can be further sub‑divided, and three dominate the current landscape: (1) Data Dependent Acquisition (DDA), which includes untargeted methodologies (shot-gun); (2) Targeted Data Acquisition (TDA) strategies (Selective Reaction Monitoring/Multiple Reaction Monitoring; SRM/MRM); and (3) Data Independent Acquisition (DIA), which includes deep scanning methods such as SWATH [1] and MS^E^ [2]. We describe these briefly below.

In a typical DDA setup, proteins are first digested (usually using trypsin) into peptides before separation and ionization. Here, two types of spectra are collected; the first (MS) based on the initial digestion, and the second (MS/MS) is based on a second round of fragmentation of selected peaks in the former. These peptide fragments are defined by 3 factors: m/z (charge state and mass), retention time and signal intensity. Identification is based on the first two factors and can be based on spectral matching against theoretical spectra (e.g., protein database) or known/annotated spectra (from a similar experiment). A second possibility is *de novo* sequencing. Fragment selection is semi-random and leads to poor reproducibility (consistency). A list of considerations has been given in Goh and Wong [3]. 

TDA requires pre-definition of the Proteins Of Interest (POI). This can be achieved by specifying the expected mass of the POI, along with the expected masses of the product fragment ions. The quantitative shift from parent mass to fragment mass is termed as a transition and can be denoted as parent mass ➔ fragment mass. The instrument repeatedly cycles and specifically screens for transitions from sample matching peptides originating from POIs. Only spectra corresponding to the same set of proteins will be screened across all samples. Throughput is an issue and only up to several hundred proteins can be monitored simultaneously but, on the other hand, TDA excels in sensitivity and quantitation accuracy. Unlike DIA and DDA, TDA does not record all transitions but only captures its intended POI signals; it is not possible to return to the data to recover additional information. This limitation means systems-wide analysis is not possible nor reversion for re-mining the original spectra. With careful POI selection, however, the specific behavior of a chosen pathway can be monitored.

**Figure 1:**
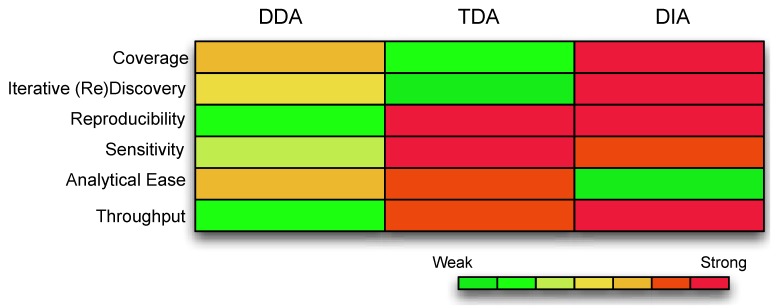
A comparison of the different features compassing each acquisition strategy. The color coding represents the strength of the data acquisition, with warmer colors as strong and cooler colors as weak. Coverage is the extent of the underlying assayable proteome. Iterative Discovery is whether the spectra can be revisited for further validations. Reproducibility refers to consistency of identifications and measurements. Sensitivity is the ability to detect low abundance ions. Analytical ease refers to whether the data is readily analyzable with minimal computational resources. Throughput refers to the number of samples that can be handled and tested simultaneously. (Abbreviations: DDA, Data Dependent Acquisition; TDA, Target Dependent Acquisition; DIA, Data Independent Acquisition).

DIA is the newest paradigm and a major driver towards true high-throughput proteomics. The basic principle is platform-driven brute-force spectra acquisition (up to several hundred are captured concurrently). Two examples of this strategy are MS^E^ [2] and SWATH [1]. In MS^E^, peptide fragments are captured within a specified m/z window [4]. SWATH, on the other hand, is characterized by repeated cycling through sub isolation windows (~25 Da apart at 100 ms each) within a specified m/z range (400–1,200) [1]. Each isolation window is also referred to as a SWATH. Unfortunately, mining DIA data is somewhat of an informatics challenge and resource intensive. At the time of writing, DIA data are still mined by predefinition of theoretical spectra from POIs in a manner similar to TDA. For a comparative summary of the 3 strategies, refer to Figure 1.

**Figure 2:**
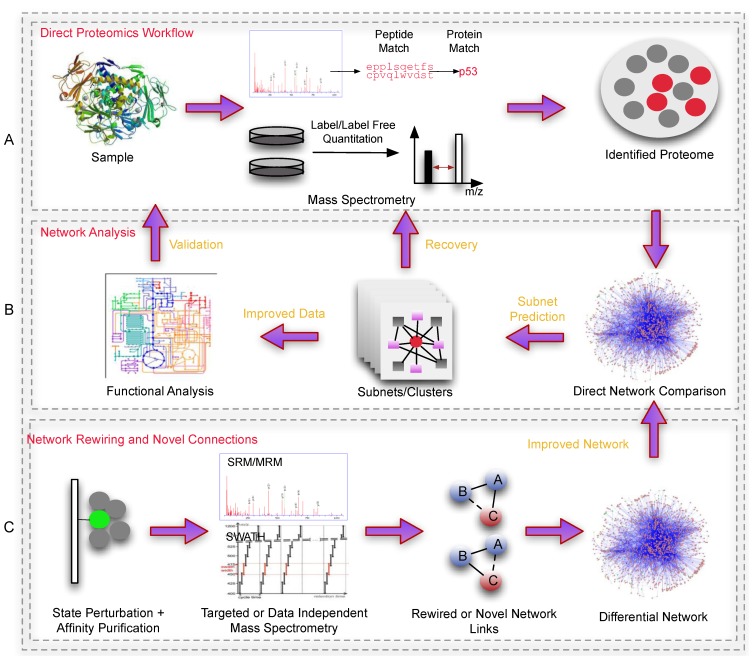
An overview outlining a general workflow using networks in proteomics. Panel A is a simple depiction of sample preparation and proteomic processing, producing a list of identified/quantified proteins. This data set normally suffers from inconsistency and coverage issues (if performed via shot-gun methods) which is difficult to analyze effectively. Panel B depicts a simple subnet-based workflow where dysregulated proteins (red) are mapped onto a network, and used to predict novel clusters. These novel clusters would consist of undetected proteins which can be re-checked (Recovery) against the original mass spectra (peptide-spectra matches that did not meet the filtering requirements). Alternatively, recovery can also be achieved via the use of SRM/MRM proteomics (not indicated). The improved data set (with expanded set of proteins), in their respective clusters (biologically contextualized) can be deployed for functional analyses. Panel C shows an example of how proteomics can improve the reference network. Here, affinity purification coupled to proteomics-based direct monitoring methods can be used to identify state-specific rewiring events or novel interactions between proteins under various conditions. These in turn, can be used to build more accurate/higher quality reference networks for biological analysis.

Protein identification and quantitation, while useful, is not fully informative about the underlying biology. Cellular biology is extremely complex and goes beyond mere quantitation of any single biological moiety. Function is achieved via interactions between molecular entities (in whatever amount they are expressed in) where they coordinate, regulate, and enforce. Of the biological entities (which includes DNA, RNA, proteins, *etc.*), the proteins play a major role in all three aspects of coordination, regulation and functional enforcement. Understanding protein function requires a systems wide analytical strategy, and is beyond the limitations of what proteomics alone can achieve. Network analysis, despite being a relatively early developing field, provides a means of contextualizing proteomics data, allowing higher-level phenotypic analysis e.g., in diseases [5]. Formally, network analysis refers to both the process of building accurate and extensive reference systems as well as devising suitable statistical and analytical strategies. High-throughput measurement platforms, e.g., proteomics, are inter-dependent with network research; the advancement of one drives the other. Today, these inter-dependencies are even greater [5]. The panels in Figure 2 show how proteomics and networks can be used in conjunction.

The integration of networks with genomics (network genomics) is a relatively well-established field. Conventional network analysis takes advantage of the larger coverage and relative stability of the measurements. But ultimately, these measurements are insufficient: RNA measurements only capture indirect information, and have little correlation with proteins levels (post-translational). Moreover, proteins are direct effectors, and their absolute quantities, to an extent (not considering the added effects of chemical modifications), determine functionality. Since proteins work directly via associations and interactions, understanding these from a systems-wide perspective is essential for understanding biology. The systems-wide study of proteins (Network Proteomics) has to improve in 2 critical areas: (1) Improving proteomics data quality, (2) Building suitable reference network libraries and analyzing them effectively. On (1), we discuss several contemporary themes that can improve data quality, which in turn will boost the outcome of downstream network analysis. On (2), we comment briefly on the rise of context-specific differential networks as well as essential issues for network analysis. 

## 2. Expanded Coverage and Consistency in Proteomics

As described earlier, all three proteomics strategies have limitations. DDA is most commonly used, but has coverage and consistency issues. TDA is of limited use beyond focused studies. Since the spectra are not captured, TDA is of limited use for direct recovery from data, although it can be used for validation and confirmation if used after a discovery-based approach. DIA potentially offers high returns on coverage and consistency but the spectra search problem presents a significant obstacle, not to mention the specific and expensive hardware requirements. 

Networks can be used to predict missing information based on existing data. For example, from DDA, critical sub-networks for which there is over-representation of detected proteins can be used for recovery of non-detected proteins. Standard/Traditional methods used include over-representation analysis (on some complex or pathway) based on the hypergeometric distribution; group-based analysis such as GSEA [6] and Functional Class Scoring (FCS) [7]; and pure networks analysis such as SNet [8] and Network Enrichment Analysis (NEA) [9]. We have already covered these approaches earlier [10]. Instead, we focus here on improving the recovery process by improving basic data quality: by building more sophisticated identification pipelines, reducing the data hole issue, circumventing search issues in large DIA libraries, and establishing “gold standards” for evaluating search algorithms and optimizing their parameters.

### 2.1. How Highly Customizable Proteomics Pipelines Can Help

Current identification software packages in proteomics can be daunting. Highly automated commercial ware from vendors remove much of the fuss and difficulty in parameter optimization and pipeline building, but may fall short in customizability and flexibility. For example, the user may be interested in combining the outputs from several non-bundled database search algorithms, including the inputs of de novo sequencing software, or integrating a functional analysis component downstream. Or perhaps the user may be interested in adapting a pipeline using existing software for non-mainstream purposes. These needs are better met by managing/combining freely available software modules, especially from the academic setting.

Unfortunately, existing software modules are scattered, and work only for specific operating platforms (UNIX, Windows, Macs), or on specific data formats (e.g., DTA, WIFF, MZML, MZXML, *etc.*). Combining these into a single workable pipeline is non-trivial.

Highly customizable pipelines can help improve protein identification such as pooling information from various peptide search engines or from several identification strategies (e.g., database search and *de novo*). These can then be scored and ranked. The high-confidence set can be used for prediction while the lower-confidence set can be used for recovery based on association to high-confidence proteins (e.g., being in the same network cluster). But these pipelines can be difficult to set up or deploy.

Without the above, one quick get-around is to divide a reference library into high- and low‑confidence databases. The former is then used as seeds for identifying network clusters based on high-confidence identifications while the latter can be used for the recovery process. This can be used within a single software environment and easily done. Using this procedure, we were able to determine which network analysis method is better at recovery based on precision and recall [11].

Several instances of customizable pipelines which are freely available include the Trans Proteomics Pipeline (TPP) [12], Proteomatic [13] and OpenMS/TOPP [14]. None of these pipelines are perfect solutions and all involve compromises at some level. For a quick summary, refer to Table 1.

**Table 1:** Pros and Cons of the various Proteomics Pipelines.

Pipeline	Pros	Cons
TPP	Very streamlined and comprehensive	Limited software options—no conversion or preprocessing; lack of expandability/flexibility or integration options
Proteomatic	Very user-friendly interface; Attempts at data integration from various platforms e.g., *de novo* and database search	Limited software options; lack of expandability/flexibility or integration options; Not as streamlined as TPP
OpenMS/TOPP	Large software options; Highly expandable/flexible and many integration options	Lack of annotation and examples; can be unstable and many software are not tested rigorously

The TPP was developed by the Institute for Systems Biology (ISB), Seattle. It is optimally developed for Windows. It acts as an integrated GUI environment that allows the user to customize the workflow from peptide identification and quantification to protein identifications. It is not able to deal with many data formats however (primarily MZML and MZXML). Proprietary formats have to be first converted via vendor’s conversion tools or via the Proteowizard suite [15]. It also does not offer a wide selection of software tools. The pipeline is based on the Peptide/ProteinProphet suite for identifications [16]. Quantitation options are also similarly limited (e.g., it only provides the software Libra for iTRAQ-based quantitation, XPRESS for calculating relative abundances and ASAPRatio for statistical assessment of relative quantitation, there does not seem to be methods for resolving absolute quantitation, which is also important). It also does not cover spectra preprocessing options, e.g. peak picking or baseline filtering. However, TPP users are free to truncate the pipeline as they see fit. For instance, peptide identifications can be performed on other software, converted to pepXML and then fed directly into TPP’s ProteinProphet. 

Proteomatic is an intuitive platform for pipeline building. It is freely available for Mac, Windows and Linux. It can be accessed using either GUI or command line. It offers utilities in two major branches: data meta-processing (under miscellaneous) and proteomics. The former allows comparisons of data sets—e.g., intersection, exclusive, union—and simple CSV manipulations. In the latter, it offers, in very specific terms, various representative software. For example, it offers OMSSA [17] under identification. For *de novo* sequencing, it offers a wrapper that can deal with PEAKs (Bioinformatics Solutions Inc., Waterloo, ON, Canada) output [18] (note that PEAKs is commercial ware), it offers a set of simple functionalities for dealing with FASTA files for library manipulation, and for quantitation, qTRACE. While it offers a more intuitive interface than TPP, Proteomatic suffers from a less streamlined/current software suite (e.g., Peptide/ProteinProphet is more current and established than OMSSA), and lack of variety and customizability. It does better at data integration since it allows *in situ* data comparisons but as of now, the integration options offered are rather basic.

OpenMS/TOPP is an open source C++ software library developed by several contributors in Germany (FU Berlin and U. Tuebingen) and Switzerland (ETHZ). It provides built-in algorithms for *de novo* identification (e.g., CompNovo) and database search (Mascot [19], Omssa [17] and X!Tandem [20], search results from other search algorithms—e.g., PeptideProphet [16]—can be converted from PepXML into idXML and incorporated directly into the OpenMS workflow). It is the most extensive of the three (provides from data conversion, feature preprocessing, to protein quantitation), but the large gamut of software options (each with multiple parameters to optimize), with generally little annotation and examples, makes it difficult to set-up. Moreover, many of the tools have not been extensively tested and it would be advisable for a newly developed OpenMS pipeline to be benchmarked against other software suites [21]. However, OpenMS is extremely promising and powerful. Recently, there is increasing work towards OpenMS pipeline building and parameter evaluation, thus allowing users to more easily develop their own workflows [21]. This suggests that workflow development should become easier as the software suite matures and the user base expands. Finally, a unique advantage of OpenMS is that it is one of the few platforms that provide support for SWATH analysis (an instance of DIA). 

### 2.2. Missing Value Imputation for Proteomics?

DDA and to some extent, TDA, suffers from inconsistency issues [22]. In extreme scenarios, up to 60%–70% of the data is incomplete (incomplete corroboration between samples or missing quantitation). This impedes proper feature selection. For example, there may be insufficient evidence to suggest whether a protein is or is not differentially expressed, leading to its mistaken inclusion (False Positive) or exclusion (False Negative). 

Moreover, a dataset with many “holes” is not amendable for many forms of statistical analysis. If the dataset is sufficiently large however, it may be possible to infer the missing values based on the distributions of what is already measured or known (Missing Value Imputation or MVI). The premise of MVI is that missing data is replaceable by some value randomly drawn from an estimate of its distribution (if known) [23].

Broadly, two factors give rise to missing values: Missing Completely at Random (MCAR) and Abundance Dependent Missing Values (ADMV) [24]. MCAR occurs stochastically due to instrument hyper-sensitivity and is independent of the peptide abundance values. ADMV occurs if the peptides are below instrument detection limit or, on the other extreme, instrument saturation. 

For individual features, performing MVI with MCAR is relatively straightforward. Techniques from microarray can be readily used. For example, simple imputation can be achieved by inputting row means, lowest observed values or just filling in with zero [25]. The first two will underestimate true biological variation while the third increases bias (towards non-detection). All three will perform worse with larger data holes. To reduce underestimation, a probability distribution can be empirically generated given sufficient observations. MCAR missing values can be randomly sampled from the former. 

More sophisticated methods use weighted averages, expectation maximization or global information for MVI. Instances include Least Squares (LS), Local Least Squares (LLS), K Nearest Neighbors (KNN) and Bayesian Principal Component Analysis (BPCA). These are discussed at length by Aittokallio [25].

ADMV is harder to resolve since observed values are unsuitable for imputation. Assuming that the peptide is below detection limit, observed values in detectable instances are unlikely representative of the unobserved and would be over-estimates if used in MVI. In the event where the peptide is actually absent, MVI would create a false positive that would skew downstream analysis.

A scan of recent literature reveals limited efforts towards solving proteomics-specific MVI. An interesting approach is that proposed by Karpievitch *et al*.; they postulated that MCAR and ADMV can be combined within a single model by estimating probabilities of the two events independently for each feature [24,26]. First, for each sample k, and a peptide j corresponding to some protein i, MCAR and ADMV are assumed to be independent. MCAR is set as a random probability π, that a peak is not observed. The chance of ADMV occurring is assumed to be the left hand tail probability drawn from the distribution N(μ_ijk_, σ_ij_^2^), that must exceed a peptide censoring function c_ij_. The probability of a peak being unobserved is given by joint probability that either MCAR or ADMV occurs. The probability of a protein being detected is given by the combined probabilities of both observed and unobserved peaks, with the restriction that all observed peaks quantities exceed a pre-specified detectability threshold. While this is an interesting approach, there are some fundamental caveats that need to be addressed: π is unlikely a fixed probability, as certain peptides are more likely to be observed than others due to instrument configuration, system sensitivity, and biochemical properties. But since the first two are fixed, an estimated π based on physico-biochemical properties may be more optimal. The second is that it is unclear if ADMV occurs purely due to signal fluctuations (and if the distribution of this fluctuation is similar to the left tail of a normal distribution) that results in non-detection. Moreover, since this modeling approach only considers signals above the threshold c, it doesn’t account for ADMV due to saturation. Perhaps modeling ADMV as a sigmoidal distribution, and defining c as a detection range (so that both long tails of the s-curve are regarded as non-detection events) may account for both possibilities.

### 2.3. Getting Around DIA Data Search Problems

DDA and TDA can be combined in tandem [27]. The findings from the former can be confirmed in the latter. Unfortunately, signal-noise, inconsistency and coverage issues in the former could mean that relevant features would be missed or buried within a sea of noise. 

Aside from improving data quality (Section 2.1), DDA-based approaches can be contextualized against biological background to yield stronger class differences or stronger features. With incomplete data, achieving proper sample stratification is difficult. However, with appropriate contextualization, this may be possible, and with it, strongly differential features between the properly stratified classes can be biologically informative. In one example, Proteomics Signature Profiling (PSP), converting protein identifications for each sample into a vector of hit rates against an ordered list of protein complexes is sufficient to recover the underlying patient subclasses and predict phenotypically relevant features, which in this case, are protein complexes [28,29]. Group-based feature identification has an improved signal-to-noise ratio. Suppose that the screen has a reliability of 50%, and protein A is identified. By itself, A’s chance of being false positive is 50%. Suppose A is part of a complex C, comprising proteins A, B, C, and E. Since B to E are not identified, the probability that all these are false negatives is (50%)^4^ = 6%. Hence, it is 8× more likely that A is false positive than B-E being missed. Conversely, if B to E are all detected, their combined false positive probability is considerably reduced (approximately 6%). In this case, even if A is missed, it is 8× more likely that the components in the complex exist against A not being detected [10]. Hence, the use of groups or “clusters” is likely to recover false negatives. We also note that using real complexes for recovery leads to significantly higher recall and precision rates than predicted ones (e.g., inferred from artificial networks) [11].

Prior to confirmation using a second platform for validation (e.g., TDA or DIA), we advocate re‑checking against the original spectra to reduce wastage on cost and experiment time. Methods from Section 2.1 are applicable. For example, the original spectra can be re-run against the larger but lower quality reference protein database (which could generate a larger set of identifications). Or undetected proteins can be checked against the full peptide list, including those lower quality PSMs (Peptide‑Spectral Matches) that may have not met the filtering criteria.

TDA for validation may be a one-time investment with limited returns since the set of POIs is small (due to platform limitations), has to be likely “correct” (based on earlier evidences), and cannot be re‑searched for future leads. On the other hand, combination with DIA can overcome the first and third limitations, while false positives can be reduced by considering only high-confidence functional groups. 

### 2.4. Gold Standards for Evaluating Protein Identifications

Given any method such as *de novo* sequencing or library search, confidence in matching spectra to peptide (Peptide-Spectra Match; PSM) can be determined by a variety of scoring measures. The *p*‑value, E value, FDR, PEP (Posterior Error Probability) and q score serve different purposes and are covered extensively in Granholm and Kall [30]. However, all these scores are dependent on deploying some form of true null, or “decoy” to estimate the false positive rate. A decoy is a theoretical sequence that should not be matchable against the MS spectra. Broadly speaking, if a spectrum maps to multiple decoys, it would possess low identification power.

A completely fair, scalable yet stringent method of generating decoys is not yet attainable. Reversing or shuffling the reference protein database may be insufficient to generate true nulls but are commonly used anyway due to ease of generation and scalability. To investigate the effect of bias further, Colaert *et al*. introduced a novel directed decoy database comprising isobaric mutated versions of the identified peptides [31]. Since the decoys are very similar to identified peptides, the ability of statistical measures to disambiguate this worst case situation can be investigated. It is disturbing that in most high-confidence identifications, a direct decoy match that scores better or equal could be found. This suggests that current decoy-search strategies need improvements despite being in heavy use and generally accepted by peer reviewers.

We need to be more certain of how accurate the peptide-to-spectra assignment is. Unfortunately, there are no gold standard data sets where all proteins, and their corresponding spectra, are known. If such gold standards existed, it would allow rigorous and tractable evaluation of the performance of each method (peptide identification or false discovery approach), and identification of the best working parameters (in a platform- or experiment-specific manner).

Despite the obvious need, this area remains a ‘blue ocean’ due to the general lack of advancements here. Noyce *et al*. recently developed MS-Spire, which simulates theoretical spectral maps based on a set of input proteins [32]. From an input FASTA file, with specified digestion parameters, proteins are converted into peptides from which their charge, mass, retention time and intensities are calculated. Unfortunately, comparisons to real data suggested much room for improvement. The authors also did not demonstrate sufficient generalizability since only one real dataset was used. Nonetheless, it is an important step in the right direction. 

Another possibility is in the form of semi-supervised learning algorithms e.g., Percolator, that discriminates between real and false PSMs [33]. A support vector machine (SVM) is trained with features from positive and negative examples. The former, arising from top PSMs, and the latter strong matches arising from matches to the decoy database. Examples of features include database search algorithm score, precursor mass error, fragment mass error, *etc*. The weighted feature vector is then used to re-rank matches from all queries, improving sensitivity. However, Percolator can potentially fail if there are insufficient good PSMs (lack of positive examples). Similarly, it could also fail if the negative dataset is improperly generated (bad choice of decoy generation, ambiguous PSMs).

### 2.5. Feature Identification

Normally, not all identified proteins are interesting. The biologist may be interested in selecting a subset of proteins for further analysis, e.g., mapping these proteins onto a network and understanding how they interact. Selection of these features can be based on some indicator of interest, typically, how aberrant the expression change is from the reference state. 

Selecting critical features from a proteomics dataset requires circumvention of its idiosyncratic limitations. Proteomics data, unlike genomics, is sparse and generally have fewer replicates. This means that conventional feature selection techniques, e.g., the standard *t*-test may not work well. Indeed, the small sample size issue, coupled with many missing values, can result in significant false positives and false negatives. To tackle this issue, more sophisticated feature-detection methods need to be deployed. In recent work, Schwammle *et al.* compared three feature selection techniques (Standard T-test, ST; Ranked Product, RP; limma/moderated *t*-test, LM; and combined limma/ranked product, LM + RP) using simulated and real data [34]. The combined approach LM + RP is promising; it outperforms all the other 3 in small sample sizes and is resilient to missing data. However, these methods have minimum requirements that need to be fulfilled: at least 1,000 features and 3 replicates (as global information is used). Or else, they tend to incorrectly estimate the *p*-values, leading to higher false positive rates. Further downstream, selected features can be further filtered based on the network e.g., dysregulated proteins that are found to be inter-connected are less likely to be false positives.

## 3. Suitable Reference Network Libraries

The issues examined in the previous section pertained to improving data quality prior to network analysis. Current knowledge on networks is still limited. To date, we are still using arbitrarily simplified network models instead of unified models (*i.e.*, containing all possible interaction types e.g., protein-protein, regulatory, metabolic, *etc*. and all possible biological entities e.g., RNA, protein, miRNA, *etc.*). 

To briefly introduce networks, these are complex systems comprising nodes linked to each other via relations. In Biology, the nodes could represent molecules e.g., protein or DNA, and the relations could be functional (e.g., protein interaction), or correlative (expressional patterns). The patterns of inter-relations can be described via various statistical measures e.g., the degree, which measures the number of connections of each node, and the cluster co-efficient, which describes the the inter‑connectivity of the neighbors of each node. In the former, highly connected nodes are commonly referred to as hubs, while nodes residing in a highly connected neighborhood are likely to be located within a cluster. Simplistically, a hub could be biologically significant as a key coordinator of function, while a cluster could correspond to a complex.

Currently, the most common instances of studied networks are the protein-protein interaction networks, or PPIN, where protein interactions are arbitrarily assayed using *in vitro* techniques e.g., yeast-2-hybrid; metabolic pathways, which are biochemically defined; and regulatory networks, where some transcription factor controls the expression of its targets, and might be itself regulated by some other factors. The choice of network types to use depends entirely on the research question. It is not uncommon to use several network types in parallel [28,35]. More importantly, there is a general shift from global network towards localized subnetwork analysis [36]. The latter approach tends to produce results that are more biologically relevant, tractable and testable. In this following section, we cover three current issues: (1) differential networks, (2) the state of cluster predictions and (3) organization of complex/cluster data.

### 3.1. The Rise of Differential Networks

A traditional approach towards network biology involves superimposition of expression data onto a reference network. This perspective is lacking in that the network is capable of rewiring during adaptation and evolution. 

While current focus is on completing the reference network, there is increasing work (both technological and computational) in understanding how the network reconfigures [37]. On the former, Bisson *et al.* [38] used affinity purification coupled to SRM (AP-SRM) to monitor quantitation changes in the binding partners of GRB2 in response to various growth signals. They demonstrated that the makeup of GRB2-associated complexes was strongly dependent on the growth factor used. Potentially, several hubs could be monitored in such a manner. But the method lacks scalability, and with SRM, the potential binding partners need be predefined. Collins *et al.* have improved the scalability by combining AP with SWATH [39]. The 14-3-3 system is rather challenging due to the large number of potential binding partners involved. They first used AP to purify the protein “preys” along with the bait proteins; the sample is then analyzed using both SWATH (library building) and shot-gun proteomics. The latter provides fragmentation and chromatographic data essential for subsequent extraction from the SWATH library. Here, they were able to analyze the behaviors of 1,967 proteins across 6 conditions with an appreciable gain in scale. Novel interactors were further tested via reciprocal “pull-down” experiments. Although such techniques still do not yield the requisite full network rewiring testing, with only a “local” range, it is worthwhile to note the extent of progress in this direction.

On the computational front, interest veers onto the other extreme, where to understand rewiring events, networks are compared globally (global alignment) using various alignment techniques. These allow a glimpse into how evolution (across species) has altered the overall network structure which in turn, provides a sense of how network reconfigurations contribute to phenomena e.g., speciation, development and disease.

Global Alignment between two networks based on pure topology is an NP-hard (Non-deterministic Polynomial time-hard) problem, which broadly means that the time required for computation increases rapidly in relation to data size. Most global alignment algorithms therefore deploy heuristics to shorten processing time. In SPINAL, Aladag *et al.* first used a neighborhood matching score method for all node pairs to create an approximate alignment [40]. This is then refined by iteratively searching for an improved solution set. Phan and Sternberg designed a more biologically relevant approach (which also greatly reduces the alignment search space) by first identifying local substructure similarities based on sequence and functional information [41]. These substructures are then condensed, and instead, only the connections between substructures are considered for the alignment. This simplifies the alignment calculation. However, the requirement (sequence and functional similarity) that defines a substructure may be arbitrary: if it is too rigid, then the alignment process may become inefficient; if too loose, information on internal rearrangements would be lost (e.g., different forms of a complex).

While many other alignment algorithms exist, we can only suggest that the choice should be based on practical concerns e.g., speed and ease of analysis, or biological relevance (at the potential cost of being more cumbersome due to incomplete annotation or a poorly studied organism). 

### 3.2. Improving Cluster Predictions from Networks

Complexes containing biologically rich information can be used to improve phenotype analysis, as well as for improving quality of data. However, complexes are compiled experimentally and annotated manually. Thus the list of known complexes is likely non-exhaustive, and does not represent the entire gamut of biological function. Since complexes contain functionally interacting proteins, we can infer that information on these is embedded within the network (e.g., PPIN). 

Most existing methods for identifying protein complexes from some network, e.g., a PPIN, are applicable only to fairly dense (but not super-dense) regions of the PPIN. These methods share a basic hypothesis that proteins in a complex have a higher degree of mutual interactions than proteins not in the same complex. An extreme case of this is CFinder, which searches for overlapping maximal cliques (*i.e.*, fully connected subgraphs) in a PPIN [42]. A more relaxed form is ClusterOne, where a cohesiveness measure was formulated to maximize similarity within a cluster and dissimilarity to its environment [43]. 

However, a significant proportion of protein complexes reside in sparsely connected regions of PPINs, and thus cannot be recovered by the approaches mentioned above. To illustrate this point, Yong *et al.* combined three yeast PPIN databases and compared the connectivity of the member components which belonged to real complexes [44]. Connectivity is a measure on a scale from 0 to 1, where 0 is defined as no connection between the member components and 1 indicates that every member is connected to each other. For yeast, which is the most complete and well-studied network, although most real complexes do reside in densely connected regions, about 10%–20% did not. This problem is further exacerbated in larger incomplete/unmerged networks, e.g., human and mouse.

One approach to recover complexes from sparse regions of PPINs is to relax the assumption that proteins in a complex should have a high degree of mutual interactions. Habibi *et al.*’s method based on k-connected subgraphs [45] is a good example of this approach. It predicts regions that are k‑connected in a PPIN to be complexes. A region is k-connected if there are at least k paths between every pair of proteins in the region. Another approach to recover complexes from sparse regions of a PPIN is to first predict the missing protein interactions so that the PPIN becomes denser, and then use some of the earlier protein complex prediction methods on this augmented PPIN [44] 

Protein complexes in super-dense regions of PPINs are also very difficult to recover by most protein complex prediction methods. In these regions, multiple complexes are highly overlapping each other. Most existing protein prediction methods would incorrectly predict the whole region as a single complex. One approach to deal with this problem is to first decompose a PPIN based on cellular location information and then run protein complex prediction on the derived PPIN for each cellular location. This approach is based on the sound hypothesis that proteins in a protein complex must be present at the same time and space to form the complex. Another idea is to first remove large hubs from a PPIN and then predict complexes from the reduced PPIN. This approach is based on the observation that such large hubs are usually found in the intersection of two overlapping complexes and their removal can greatly reduce the number of connecting edges between in two complexes in the PPIN. Liu *et al.*’s method is an example that employs both of these ideas [46]. Another approach is to decompose a large predicted protein cluster, which is likely to be a merger of multiple complexes, using the knowledge that certain protein-protein interactions are mutually exclusive, e.g., two proteins compete for binding to a third protein, and thus cannot simultaneously exist within the protein complex [47].

### 3.3. Organized and Representative Cluster/Complex Repositories

Biological complexes provides the best biological reference for recovery and validation of undetected proteins [11]. Information on biological complexes however is limited. Currently, the most extensive resources on biological complexes are found in manually curated databases, e.g., CORUM (human) [48] and MIPS (yeast) [49]. 

The gamut of representative biological function may not be fully available to test every biological scenario, e.g., cardiovascular and neurological. Hence, it is essential to boost the set of complexes either via prediction (from a network), or experiments (e.g., AP-MS).

Certainly these complexes will also need improved functional annotation and organization. Currently, functional annotation is provided via a systematic organization of biological terms referred to as Gene Ontology (GO) [50]. Proteins within a complex are likely to share common GO terms but these terms can be numerous, similar in meaning to each other, or too general. It is thus essential to fine-tune granularity of GO terms by (1) accepting only functions that are enriched within the group, and (2) reducing the redundancies between terms [10]. It is also worthwhile to organize the complexes to reduce redundancies within or between databases. Wu *et al.* investigated this by reducing redundancies within CORUM [41], HPRD [51] and PINdb [52], as well as in their combined version, CHPC2012 [53]. In their approach, a significance score was determined for each protein complex based on functional enrichment. Protein complexes are then compared pairwise based on score similarities. If comparison yields similarity better than a predetermined threshold, then the complexes are either merged, or the lower scoring discarded. 

A second level of organization is to further sub-group the complexes into those with related functions (complex group) that are distal from other complex groups. GO itself is an hierarchical network of biological terms with a tree structure, where the root term is least specific, and leaf terms very specific. Terms that are very far apart on the tree are likely to relate to very different biological functions. Hence, by determining the relative term path distances between complexes, it is possible to organize them into groups of functional relatedness. Moreover, it should be expected that if a given complex is found to be significant, then it follows that the other complexes within the same group (due to relatively closer related functions) are also likely to be affected, and should be prioritized for investigation. 

## 4. Conclusions

Just as networks can be used to improve proteomics analysis, the reverse is also true; some requirements from proteomics would render the data better for downstream analysis while others require advances from network biology.

Currently, both areas are rapidly evolving and changing. Here, some promising and noteworthy developments likely to have profound impact in the future have been described. It may be possible that DIA methods will eventually supercede DDA and TDA approaches in the near future, however, in order for that to happen, enormous leaps and advances in protein derivation and informatics analysis first need to be achieved.
